# Validating viral quasispecies with digital organisms: a re-examination of the critical mutation rate

**DOI:** 10.1186/1471-2148-5-5

**Published:** 2005-01-15

**Authors:** Iñaki Comas, Andrés Moya, Fernando González-Candelas

**Affiliations:** 1 Institut Cavanilles de Biodiversitat i Biologia Evolutiva. Universitat de València. Spain

## Abstract

**Background:**

In this report we re-examine some recent experiments with digital organisms to test some predictions of quasispecies theory. These experiments revealed that under high mutation rates populations of less fit organisms previously adapted to such high mutation rates were able to outcompete organisms with higher average fitness but adapted to low mutation rates.

**Results:**

We have verified that these results do hold in the original conditions and, by extending the set of initial parameters, we have also detected that the critical mutation rate was independent of population size, a result that we have found to be dependent on a different, contingent factor, the initial fitness vector. Furthermore, in all but one case, the critical mutation rate is higher than the error threshold, a key parameter in quasispecies theory, which prevents its extrapolation to natural viral populations.

**Conclusion:**

From these results we conclude that digital organisms are useful tools for investigating evolutionary patterns and processes including some predictions from the quasispecies theory.

## Background

RNA viruses are among the most infective pathogens affecting plants, animals and humans. Several of their features such as their reduced genomes, high genetic heterogeneity, large population sizes, short generation times and fast evolutionary rates place them among the best models for evolutionary and population genetic studies [1,2]. These same features explain why they are so difficult to eradicate. Many of them are able to establish chronic infections because their high mutation rates allow them to escape from the immune system pressure.

As a consequence, selection, that translates in competition with the host and among viral variants, usually results in the persistence of the most infective, pathogenic or more persistent variants. The molecular bases for this genetic variability are three mechanisms differentially used by each kind of virus: mutation, homologous and non-homologous recombination and genome rearrangement [3].

Attempts to model the evolutionary dynamics of RNA viruses incorporate their most relevant features, such as large population sizes (due to their short replication times RNA viruses can reach population sizes of around 10^10^individuals in short times), high mutation rates (in the order of 0,1–1 mutations per genome and replication round (m/g/r) derived from lack of proof-read correction in the polymerase), and small genome sizes (ranging from 3 to 30 kilobases).

For years RNA virus population dynamics has been studied under the classical population genetics framework [1], thus allowing the development of models that explained their evolution in terms of selection, mutation, genetic drift and, less importantly, migration within and among hosts. Under this framework theoretical predictions such as the Red Queen hypothesis [4], frequency-dependent selection [5,6] or clonal interference [7] have been demonstrated with experimental populations of viruses.

Despite these achievements in the late 70's some results suggested that the evolution of RNA viruses might be better explained by a quasispecies model. The quasispecies concept was formulated by Eigen [8] in his studies on the evolution of the first replicons. The concept arises as an alternative to the neutral theory [9] which requires small population sizes and large genomes. A population of replicons with these characteristics cannot explore the whole neutral space of an adaptive landscape and, consequently, the stochastic differentiation of the molecules is possible. But in the case of molecules with small genomes and large population sizes (such as early replicons) the whole neutral space can be explored thus avoiding the effect of the genetic drift. This property along with high mutation rates allows quasispecies formation in viral populations. A quasispecies has been defined as a cloud of mutants organized around one or a few high fitness variants and with very low Hamming distances among them. The high mutation rates are the connective agent between the members of the quasispecies, with their frequencies depending on their replication fidelity and that of the rest of neighbor mutants. This mutational coupling implies that the object of natural selection is the quasispecies as a whole and not each individual variant. The quasispecies structure has three important implications [10]:

- Selection acts upon the quasispecies as a whole and not upon individual variants. The result is that under appropriate conditions lower fitness variants can outcompete higher fitness ones (survival of the flattest vs. survival of the fittest).

- Genetic drift has no relevant effects: their tiny genomes, large population sizes and high mutation rates allow the exploration of all the neutral space around the master sequence.

- The average consensus sequence remains stable along quasispecies evolution.

This model contrasts sharply with conventional population genetic models in which the existence of a large number of neutral mutations would lead to genetic drift of the population and the individual is the unit of selection rather than a cloud of related variants [11]. This last difference is most relevant when quasispecies theory is applied to real entities, such as RNA viruses, in two contexts. First, RNA viruses represent the vast majority of emerging pathogens and there is a growing interest in the application of evolutionary principles for the control, prevention and treatment of diseases caused by them [2]. Second, RNA viruses represent the best example of measurably evolving populations [12] and as such are widely used to experimentally test many postulates of evolutionary theory [2,13]. Hence, differences on the nature of the unit of evolution in RNA viruses may have important consequences in practical applications and experimental verification of evolutionary theory.

The difficulty in experimentally testing some predictions of quasispecies theory has led to the search of alternative systems. In this work we have used digital organisms as an approximation to the population dynamics of RNA viruses. Digital organisms are self-replicating entities and compete for access to resources, in this case CPU cycles, as implemented in the AVIDA platform [14,15].

Avidians are programs (genomes) composed by arrays of logical instructions (genes) that allow them obtaining CPU cycles. There are 28 possible instructions. The number of instructions in a digital organism is equivalent to the genome size in a biological organism [16,17].

Many studies have been done using the AVIDA platform. The possibility that genome sizes change during the course of evolution and the rewards that they can obtain by the combination of functions have allowed investigations about the evolution of genomic complexity [18]. Furthermore the possibility of studying their evolution throughout long periods of adaptation and competition has allowed the reproduction of studies originally performed with other asexual organisms such as viruses and bacteria [19]. Other studies have attempted to determine the effect of each possible mutation on the fitness of a genome and the nature of their interactions [20,21].

In this work we have focused in a recent study by Wilke et al. [22] with digital organisms in which they concluded the validity of one of the principal tenets of quasispecies theory. The prediction is that less fit organisms can outcompete fitter organisms when mutation rates are high. The dynamics of their experiments consists of generating, from a common ancestor, pairs of organisms adapted to high (lower fitness organisms) and low (higher fitness organisms) mutation rates. Then, competition experiments between high and low fitness organisms are performed at different mutation rates. As the mutation rate increases, these experiments result in the winner being always the lower fitness variant. This indicates that previous adaptation to high mutation rates generates less fit but very robust variants. Therefore, under high mutation rates these variants generate a better adapted cloud of mutants. On the other hand, high fitness variants are in higher but steeper adaptation peaks and in consequence are more sensitive to mutation. Therefore at high mutation rates there is *survival of the flattest *and not *survival of the fittest*.

Here we have extended the original experimental conditions in order to study the effect and interaction between three of the key factors in the quasispecies model: population and genome sizes and mutation rates. Our results indicate that chance events in the form of historical contingency play an important role in the evolution of these populations. Moreover we have established a new, corrected mutation rate necessary for quasispecies formation with a higher value than the original one. The implications of this correction are discussed.

## Results

We considered three factors affecting the critical mutation rate in our digital organisms: genome size, population size and the influence of the initial fitness vector. This is a vector of randomly assigned priorities for the first time evaluation of each organism fitness, incorporated to prevent the system from collapsing if all organisms simultaneously try to enter the CPU, and can be interpreted as a historical, contingent factor in evolution. The range of genome sizes studied varied from 54 to 272 instructions (Table 1). Our exploratory experiments indicated that the initial fitness vector might have an important effect on the results of competition between pairs of organisms adapted to low and high mutation rates. This was most apparent when comparing results using the original, fixed initial vector used by Wilke et al. [22] for all the competitions involving the same pair of organisms and those obtained when the initial fitness vector was a random one, with different values for each experiment. Hence, in the original study for 3600 individuals the critical mutation rates varied between 0.88 and 3.66 with a mean value, normalized according to our criterion for estimating the critical mutation rate, of 1.386 (standard deviation, SD = 0.777). In our experiments for this same population size and random initial vectors, critical genomic mutation rates ranged between 0.5 and 3 but with a higher average value, 2.045 (SD = 0.757). Consequently, we decided to proceed with two series of experiments, one using the same initial fitness vector for all the competition experiments for each pair of organisms and the other with initial fitness randomly assigned in each competition.

**Table 1:** The twelve digital organisms used in the experiments. Size reflects the number of instructions in the corresponding genomes (genome size).

Organism	Size
C185	54
C212	62
C148	70
C119	86
C280	90
C238	92
C216	96
C149	108
C202	134
C295	207
C274	241
C222	272

### Fixed initial fitness vector

Table 2 shows the critical rates for organism and population size in the experiments with the same, fixed initial fitness vector for each pair of organisms. Only population sizes equal or lower than N = 3600 individuals were assayed, since the original experiments involved only 3600 individuals. Hence, it was impossible to assign the same initial fitness vector used by Wilke et al. [22] to larger population sizes. In three of the twelve competitions (Table 2) we encountered some differences with respect to the critical rate calculated by Wilke et al. [22]. In organisms C202 and C149 this rate was smaller (1.75 and 0.5 instead of 2.25 and 0.88, respectively) and larger for organism C238 (1.25 instead of 0.88). The remaining rates are equal to those obtained by Wilke et al. [22] and the differences are due to the better approximation obtained in the original paper through some extra experiments. In our case these additional experiments were not performed because we were more interested in comparing the rates between the two parts of our study.

**Table 2:** Critical mutation rates using one fixed vector per organism. The corresponding values obtained by Wilke et al. [22] for 3600 individuals are shown in the last column ("Original").

	Population size					
	
Organism	250	500	1250	2500	3600	Original
C185	1.25	1.25	1.25	1.25	1.25	1.13
C212	1.25	1.25	1.25	1.25	1.25	1.13
C148	0.75	0.75	0.75	0.75	0.75	0.88
C119	1.75	1.75	1.75	1.75	1.75	1.75
C280	1.25	1.25	1.25	1.25	1.25	1.13
C238	1.25	1.25	1.25	1.25	1.25	0.88
C216	1.25	1.25	1.25	1.25	1.25	1.25
C149	0.5	0.5	0.5	0.5	0.5	0.88
C202	1.75	3	1.75	1.75	1.75	2.25
C295	1.75	1.75	1.75	1.75	1.75	1.88
C274	3	3	3	3	3	3.6
C222	3	3	3	3	3	3.6

### Random initial fitness vector

**Figure 1:**
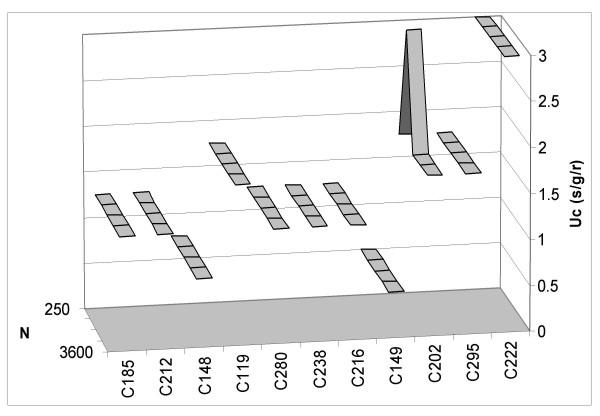
**Critical mutation rates using one fixed initial vector per organism. **Critical mutation rate (U_c_) versus population size (N) for each organism used in the experiments with one fixed initial fitness vector per experiment. In order to obtain exact replicates of the original simulation [22] we did not included population sizes larger than N = 3600.

As expected from our preliminary results, the use of an initial random vector for each experiment and not for each organism resulted in clear differences with the results encountered by Wilke et al. [22]. These differences are shown in Table 3, which presents a summary of the critical mutation rates obtained for each organism and population size using one random vector in each experiment and those originally with one fixed initial vector and N = 3600. In eight of the 11 cases studied the critical rate was higher than the original value. Only in two cases this value was equal to the one originally reported by Wilke et al. and in one case, for organism C222, it was lower. Table 3 summarizes the critical mutation rate encountered for each organism and population size (see also Fig. 1). The correlation between the critical mutation rate and population size for each organism allows the separation of the twelve organisms in three main groups (Table 4): (i) those with a significant, positive correlation rate (C212, C148, C119, and C202); (ii) organisms with no significant correlation (C185, C222, C280, C149, C216 and C295), and (iii) organism C238, which is the only one with a significant, negative correlation rate. Organism C274 was excluded from this analysis because it did not show a clear pattern of fixation.

**Table 3:** Critical mutation rates using one random vector in each experiment. The last column ("Original") presents the results obtained by Wilke et al. [22] for 3600 individuals and one fixed vector in all the experiments with each organism.

	Population size							
	
Organism	250	500	1250	2500	3600	6400	10000	Original
C185	2.25	2.25	2.25	2.25	2.25	2.25	2.25	1.13
C212	0.5	0.5	1.25	1.25	1.25	1.25	1.25	1.13
C148	0.5	0.5	1.25	1.25	1.75	1.75	1.75	0.88
C119	0.5	0.5	1.75	1.75	1.75	1.75	1.75	1.75
C280	1.75	2.25	1.75	2.25	2.25	2	2.25	1.13
C238	2	2	1.75	1.75	1.75	1.75	1.75	0.88
C216	3	3	3	3	3	3	3	1.25
C149	1.5	2	2.25	1.75	2.25	1.75	2	0.88
C202	0.5	0.5	0.5	0.5	2.75	2.75	2.75	2.25
C295	2.25	2.25	3	2.25	3	2.75	2.75	1.88
C274	No pattern							3.6
C222	0.5	0.5	0.5	0.5	0.5	0.5	0.5	3.5

**Table 4:** Correlation between population size and critical mutation rate in digital organisms. Correlation coefficients (r) were calculated from the experiments with one random vector in each case (Table 3). An asterisk indicates a significant difference from r = 0 for α = 0.05. Two asterisks indicate a significant difference after Bonferroni's correction (α' = 0.0045).

Organism	r	P-value
C185	Constant	
C212	0.791	0.034 *
C148	0.932	0.002 **
C119	0.791	0.034 *
C280	0.487	0.268
C238	-0.791	0.034 *
C216	Constant	
C149	0.277	0.547
C202	0.866	0.012*
C295	0.552	0.199
C274	Not applicable	
C222	Constant	

**Figure 2:**
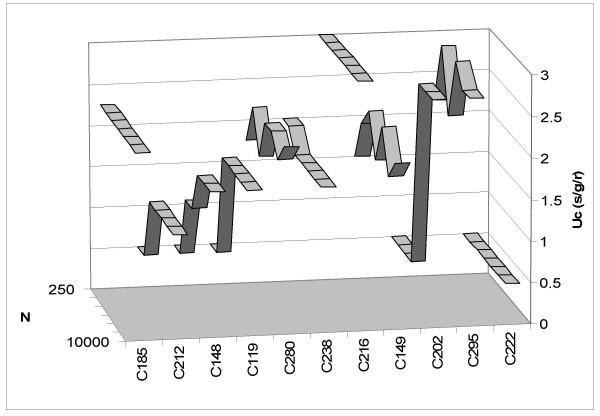
**Critical mutation rates using random initial vectors per organism. **Critical mutation rate (U_c_) versus population size (N) for each organism used in the experiments of one random initial fitness vector per organism.

Nevertheless, despite these differences between organisms we cannot conclude that there is a globally significant effect of population size on critical mutation rate. Using Bonferroni's correction for multiple comparisons we obtained a new significance level α' = 0.0045. Therefore only organism C148 has a significant, positive correlation (r = 0.932, P = 0.002). But the differences in the use of a random or fixed initial fitness vector are clear and can be observed by comparing Figures 1 and 2 where values of the critical mutation rate for each organism according to population size are represented. It seems that the use of one fixed initial vector per organism reduces variability in the results.

**Figure 3:**
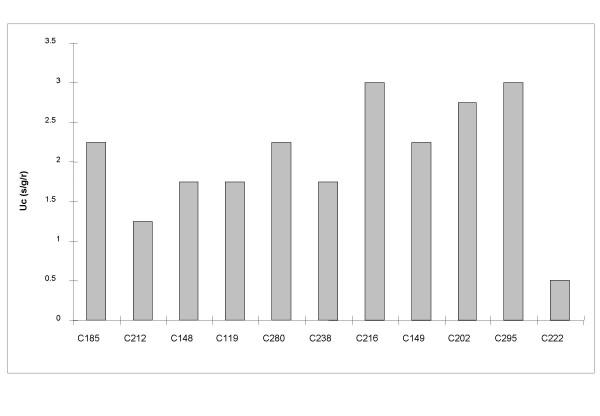
**Genomic mutation rates necessary for quasispecies formation **for each of the eleven digital organisms with a population size N = 10000.

Table 5 shows the critical mutation values found for a population size of 10000 individuals. It can be observed that there is no correlation between critical mutation rate and genome size (r = -0.269, P = 0.424). For comparison, we also compiled similar data for RNA viruses (Table 6), including retroviruses, and we did not encounter a significant correlation (r = 0.636, P = 0.125) between genome size and mutation rate (Fig. 3).

**Table 5:** Population size and critical mutation rate in digital organisms. Correlation (r = -0.267, P = 0.428) between critical mutation rates (U_C_) calculated for a population size of N = 10000 individuals and genomic size of digital organisms.

Organism	Size	U_C_
C185	54	2.25
C212	62	1.25
C148	70	1.75
C119	86	1.75
C280	90	2.25
C238	92	1.75
C216	96	3
C149	108	2
C202	134	2.75
C295	207	2.75
C284	241	N.A.
C222	272	0.5

**Table 6:** Population size and critical mutation rate in viruses. Correlation (r = 0.636, P = 0.125) between the experimentally calculated genomic mutation rate (μ_g_) and genomic size of some RNA viruses (adapted from [35]).

Virus	Size (kb)	μ_g_
Lytic RNA viruses		
VSV [27]	11.2	1.07
Poliovirus [36]	7.4	0.81
Influenza A virus [36]	13.6	0.99
Retroviruses [37]		
Spleen necrosis virus	7.8	0.16
Molony murine leukemia virus	8.4	0.029
Rous sarcoma virus	9.3	0.43
HIV-1 [38]	9.2	0.22

## Discussion

The quasispecies model requires a series of conditions to be fulfilled. These requirements are related to four key factors: population size, mutation rate, genome size and neutrality [11,23]. In our study with digital organisms we have analyzed three of these factors and we have related them to known results in virus evolution. The three main conclusions derived from this study are:

1) The use of different initial fitness vectors for otherwise identical experiments results in unpredictable effects on critical mutation rates for different population sizes. These effects were not detected in the original experiments by Wilke et al. [22] and can alter their conclusions, as contingency, or historical factors, are introduced in the system through different initial conditions leading to different final outcomes.

2) There is no significant correlation between genome size and critical mutation rate.

3) The originally calculated critical mutation rates underestimate their real values.

Despite the lack of a general correlation between critical mutation rate and population size, the comparison of Figures 1 and 2 reveals a clear difference with the initial study. By using one fixed initial fitness vector per organism, Wilke et al. [22] eliminated variability in the outcome of competition (Fig. 2). Our study results in different individual responses to changes in population size. In fact, only three of the organisms analyzed maintained a constant response to these changes when different random vectors were used in each of the experiments. Therefore it will be interesting to analyze why certain organisms are more strongly influenced by population size than others. Under the quasispecies model it is expected that increasing population size will favor the establishment of a quasispecies [11]. A large population size allows the exploration of the neutral space that surrounds the master sequence hence avoiding the effects of genetic drift. If a correlation between the two factors is to be expected, then it should be negative, as with larger population sizes a lower mutation rate is needed to maintain the equilibrium quasispecies structure. Nevertheless, theoretical and simulations results by Wilke et al. [22] indicate that this is not a true correlation but a phase transition as at low population sizes the critical mutation rate becomes more difficult to ascertain. Our results do not allow to discriminate between both alternatives but they show substantially more variability among organisms (Fig. 1) than the ones reported by Wilke et al. [22], hence pointing at a more complex scenario than that depicted in a simple phase transition.

Another key factor in the quasispecies model is genome size. The establishment of the quasispecies is easier in populations with small genomes, as in these the number of neutral sites is reduced and therefore the neutral space is also smaller. However the relationship between critical mutation rate and genomic size to be expected is somewhat contradictory. On the one hand, larger genomes need higher mutation rates because the neutral and adaptive landscapes are larger. On the other hand, it is well known that large genomes require a stability not supplied by high mutation rates, hence the existence of an error threshold that will be discussed later. In fact Eigen [24] proposed that there should be a negative correlation between these two factors. However in our analyses we have found no such correlation neither in digital organisms nor in experimental data with RNA viruses (Tables 5 and 6), in agreement with [25]. However the absence of a significant correlation does not necessarily mean that there is no relationship between the two factors. Genomic mutation rates impose a limit on the maximum genome size but this does not imply that the best adaptive strategy is to reach the maximum variability attainable for the corresponding genome size [26].

Our correction to the critical mutation rates estimated in the original paper relates directly to the limits imposed by the mutation rate. In most cases Wilke et al. [22] obtained critical mutation rates larger than 1 (between 1.13 and 3.5). However, in our experiments we have found these rates to be even larger. This correction in the mutation rate needed for the establishment of a quasispecies is important because estimates of genomic mutation rates of RNA viruses are usually about or below 1 [27,28] (Table 6). This limit is known as the error threshold and is another key concept for quasispecies theory. It represents the mutation rate beyond which the information in the molecules would be lost due to degeneracy. The critical mutation rates obtained in the vast majority of cases here reported are larger than 2. If these values were similar in "real" virus populations then they would be beyond the error threshold and therefore the viral quasispecies would not be possible. Therefore, it is important to determine up to which point the comparison of mutation rates between viruses and digital organisms is valid. There are two extreme possibilities: either it is not valid, and therefore digital organisms cannot be invoked as a proof of the evolution of RNA viruses as quasispecies, or if the analogy is possible this means that, at least in the case of RNA viruses, the quasispecies is a theoretical possibility but the practical conditions needed are not met. The presence of an error threshold in viruses is a consequence of a trade-off between the maximization of variability (genomic mutation rate) and the maintenance of molecule integrity (genomic size). It is this trade-off, translated into an error threshold, which might prevent virus quasispecies formation. In this way, the error threshold would not be proof of their existence [29] but rather of their impossibility in RNA viruses.

In conclusion, although some predictions from quasispecies theory are not fulfilled in our experiments, we do have observed the principal prediction that lower fitness competitors can win the competition to high fitness ones, but only under very high mutation rates. Recently, several papers [11,23,30] have pointed out the possibility that RNA viruses do not meet all the requirements for quasispecies persistence. The results from this study also suggest that the necessary mutation rates are not attainable either. One possible explanation is that viruses are necessarily more constrained in their evolution than digital organisms. Some experiments demonstrate that the variability found in natural isolates of RNA viruses is not correlated to their mutation rate because some form very conserved RNA secondary structures [31]. Similarly, it has been demonstrated the frequent selection of the same mutations in the HIV *gag *region in isolates from different patients, an indication of the limited adaptive solutions able to produce escape mutants to the immune response of cytotoxic T lymphocytes [32]. Further restrictions could be related with the mechanisms and routes of virus infection [33].

Analogies are very useful in science, but they have to be used cautiously. Similar features and dynamics between digital organisms and RNA viruses are tempting and usually lead to conclude that both kinds of entities are governed by the same laws. This is not necessarily the case, as practitioners of the comparative method know. In any case, digital organisms are an extraordinary system to experiment with controlled, repeatable evolution conditions and further work with them is necessary to ascertain which evolution features are of their own and which are of common application to other evolving entities.

## Methods

### Experimental design

The project was started with the twelve pairs of organisms generated in a previous experiment [22] that had been adapted to two different mutational regimes. The 12 ancestral organisms originating each of the twelve pairs were adapted to low mutation rates (0.5 mutations/genome/replication round – m/g/r) and to high mutation rates (2 m/g/r) for 1000 generations. In all the cases the organisms adapted to a low mutation rate, denoted A, had a significantly larger fitness than their corresponding pair, adapted to a high mutation rate and denoted B.

With these twelve pairs we followed the same experimental procedure designed by Wilke et al. [22]. Basically, we placed in competition equal numbers of A and B organisms during 50 generations. Unlike the original experiment, we did not restrict to a single population size (N = 3600) but we added four smaller (N = 250, 500, 1250, 2500) and, when possible, two larger (N = 6400 and 10000) sizes. The mutation rates under which the competitions were performed were 0.5, 1.0, 1.5, 2.0, 2.5 and 3.0 m/g/r. The A organisms carried a label such that we could follow their proportion in the population.

### Initial fitness vector

In AVIDA organisms occupy the limiting environmental resource, the computer CPU, depending on their "fitness". In order to prevent the collapse of the system when all the competing organisms simultaneously try to use the CPU, there exists one feature designed to prevent the simultaneous replication of all organisms at the start of the competition, when all the organisms might be equally fit since they have not been tested yet in the environment. This is achieved by asynchronously introducing organisms in the competition system by assigning an initial fitness to each organism that introduces a small time lag in the accession to the CPU. For this, Wilke et al. [22] generated an initial fitness vector in the population for each pair of competing organisms. This vector was generated at random and assigned a different initial fitness for each of the 3600 individuals in the original competition. All the experiments for each pair of organisms were carried out with the same initial fitness vector.

During exploratory experiments we noticed that this vector could play a decisive influence in the result of the competition. In consequence, we divided the study into two parts. In the first one we kept the vector assigned by Wilke et al. [22] to each pair of competing organisms, and we adapted it for other population sizes whenever possible (N = 250, 500, 1250, 2500 individuals). On the other hand, for all the population sizes (including N = 6400 and 10000 individuals) we generated a different random initial vector for each experimental replicate. Therefore, for this second part we generated 252 distinct vectors for pair of organisms in contrast to the five (one per population size) generated in the first part of our study or the single one generated by Wilke et al. [22] for N = 3600.

### Critical mutation rate determination

The critical mutation rate is "the midpoint between the highest rate at where A prevailed and the lowest rate where B prevailed" [22]. It represents the rate at which the quasispecies effect is important. We measured this critical parameter as the average of the two rates at which a shift in the winner was observed.

It is necessary to clarify the conceptual difference between the critical mutation rate and the error threshold. The first one is the rate at which the prediction of quasispecies theory that organisms with lower fitness can win the competition is fulfilled. However the error threshold is the genomic mutation rate beyond which the information in the molecules that compose the quasispecies loses sense due to mutational degeneracy [29]. In practical terms, this means that this is the maximum rate that the virus can support. The relationship between the two rates is clear: the critical mutation rate must be necessarily lower or equal than the error threshold because otherwise the quasispecies effects cannot be measured.

### AVIDA configuration

We used versions 1.4 and 1.6 of the AVIDA program. Basically, digital organisms are chains of instructions that act over the CPU with the objective of reproducing as fast as possible. In this manner the CPU time becomes the limiting resource in their evolution. During replication their genomes can mutate and, as a result, a system with variation and therefore with selection and evolution is obtained. Genome sizes of the twelve pairs of digital organisms varied between 54 and 272 instructions (Table 1).

AVIDA works with some input files that determine the characteristics of the world during the population's evolution. In this case we used the "COPY_MUT_PROB" in the "GENESIS" file, which is the mutation rate that results from dividing the genomic mutation rate by the genome size. In the "EVENT_LIST" file we specified the order of introduction of the individuals and marked each with a hereditary label (A = 1, B = 0). Generally 50 generations were enough for the fixation of the A or B organism in the population. Configuration files used in these experiments are available from the authors web site [39].

### Statistical analysis

Each mutation rate-population size combination was replicated six times. For the verification of the relation between the population size and the critical mutation rate we used Pearson's correlation coefficient and a significance level of 5% using Bonferroni's correction [34]. The same analysis was used for the correlation between genomic sizes and critical mutation rate. Both analyses were carried out with SPSS 11.0 (SPSS Inc.).

## Authors' contributions

IC performed all the simulations, made the statistical analyses and wrote the first draft of the manuscript. AM contributed to the design of the experiments and the discussion of the results. FGC designed and supervised the experiments, discussed the results and their analyses and wrote the final version of the manuscript. All the authors read and approved the final manuscript.
